# Prediction of Chronic Kidney Disease Based on Simulated Serum Analysis by Vibrational Spectroscopy

**DOI:** 10.3390/bios16060347

**Published:** 2026-06-21

**Authors:** Diogo Serrano, Paulo Zoio, Luís P. Fonseca, Cecília R. C. Calado

**Affiliations:** 1 iBB—Institute for Bioengineering and Biosciences, i4HB—The Associate Laboratory Institute for Health and Bioeconomy, IST—Instituto Superior Técnico, Universidade de Lisboa, Av. Rovisco Pais, nº1, 1049-001 Lisbon, Portugal; diogosserrano@gmail.com (D.S.); pjzoio@gmail.com (P.Z.); luis.fonseca@tecnico.ulisboa.pt (L.P.F.); 2 ISEL—Instituto Superior de Engenharia de Lisboa, Instituto Politécnico de Lisboa, R. Conselheiro Emídio Navarro nº1, 1959-007 Lisbon, Portugal

**Keywords:** kidney diseases, IR spectroscopy, biomarkers, monitoring

## Abstract

The development of new technologies enabling rapid, frequent, and reagent-free monitoring of kidney function is recognized as being of paramount importance. In this work, mid-(MIR) and near-infrared (NIR) spectroscopy were compared for the prediction of key renal biomarkers—creatinine, urea and albumin—using 54 serum solutions mimicking the biochemical profiles of five stages of chronic kidney disease (CKD). MIR spectra were acquired in a high-throughput microplate platform after a simple dehydration step, while the NIR spectra were obtained directly from liquid serum using a fiber optic probe. After evaluating several spectral pre-processing methods and targeted spectral regions, excellent regression models (R^2^ > 0.9 for the best models) were obtained for the three biomarkers. MIR provided highly accurate urea predictions, whereas optimized NIR sub-regions enabled excellent estimation of creatinine and albumin. Both MIR and NIR, associated with supervised classification methods, enabled us to successfully distinguish healthy from diseased profiles and to identify the diseases state with AUC > 0.93. These findings highlight the complementary value of MIR and NIR spectroscopy for kidney disease assessment and their potential integration into point-of-care diagnostic systems.

## 1. Introduction

Chronic kidney disease (CKD) is a highly prevalent and steadily growing global health problem, affecting roughly one in 10 adults and driving excess cardiovascular morbidity, premature mortality, and escalating healthcare costs. Its progressive, often asymptomatic course, organized into five KDIGO stages [1], leads to diverse individuals being only identified as presenting CKD once substantial and irreversible nephron loss has occurred, narrowing the window for renoprotective intervention. The WHO recognizes that approximately 674 million people live with CKD, and that kidney disease is one of the fastest-growing causes of death globally, being projected to become the fifth leading cause of death by 2050, with a predicted 33% increase in age-standardized death rate and a 28% increase in age-standardized disability-adjusted life years (DALYs) [2]. This urges society to invest in health systems to integrate prevention, early detection, and management of kidney disease into national health policies. However, conventional assessment of kidney function relies on intermittent measurement of serum creatinine (for the Estimated Glomerular Filtration Rate, eGFR) and urea, that unfortunately are obtained by laboratory-based chemistry analyzers, that consequently are centralized, resource-intensive, and poorly suited to high-frequency or decentralized monitoring. In our previous study [3], we outlined this diagnostic gap and pointed out that mid-infrared (MIR) and near-infrared (NIR) spectroscopy can provide rapid, reagent-free quantification of creatinine, urea, and albumin in a fetal bovine serum matrix using harmonized chemometric pipelines, highlighting their potential to underpin point-of-care (POC) solutions for CKD screening and follow-up.

Within this context, there has been growing interest in exploiting vibrational spectroscopy—both MIR and NIR—for kidney-related diagnostics. MIR spectroscopy, by probing fundamental vibrational modes, offers chemically specific bands for urea, proteins, and lipids, while NIR spectroscopy, based on overtones and combination bands, can be directly applied to aqueous matrices using fiber-based probes. These attributes have motivated a range of proof-of-concept studies across diverse matrices: aqueous and buffer systems have been used to quantify urea or albumin and to optimize wavelength selection for potential sensors, for example, in multi-wavelength NIR/diode-laser approaches to monitor urea in aqueous films [4] and in PBS-based NIR work on human serum albumin [5].

Urine has been extensively explored as a simpler and non-invasive diagnostic matrix, with classic NIR spectroscopic studies enabling the quantitation of urea, creatinine, and protein, and subsequent work extending to multicomponent panels including glucose, ketones, and proteins. Analogous MIR and ATR-spectroscopic strategies have quantified urinary analytes or extracted spectral biomarkers from dried urine films [6]. Although urine is attractive as biofluid because it is non-invasive and easy to collect, urine-only approaches have important limitations for CKD diagnosis/staging when compared with blood/serum. Urinary readouts are strongly affected by pre-analytical and biological variability (hydration/dilution, diurnal variation, diet, exercise, intercurrent illness/UTI, and sample handling) [7], and even ratio metrics such as the urine albumin–creatinine ratio (uACR) require standardized collection (preferably first-morning void) [8] and confirmatory repeat testing because spot measurements can vary substantially day-to-day. Moreover, albuminuria is not universal in CKD (e.g., non-albuminuric phenotypes, particularly in diabetes and vascular CKD) [9,10], so urine-based screening alone can miss clinically relevant reductions in kidney function. By contrast, serum/blood biomarkers underpin the central clinical axis of CKD evaluation—estimated GFR from serum creatinine (and/or cystatin C) together with albuminuria—providing a more direct and standardized window into systemic solute accumulation and glomerular filtration decline, which is also advantageous for constructing calibration sets intended to emulate CKD progression.

More recently, label-free MIR or NIR has been applied to dialysate for online hemodialysis monitoring [11], where matrices are compositionally constrained, and to saliva or serum for CKD screening and phenotyping. ATR-MIR and related MIR-based studies have shown salivary spectral signatures and urea bands that discriminate CKD from controls and suggest saliva as a non-invasive CKD POC fluid [12]. In parallel, Navarro-Esteve et al. [13] evaluated ATR-MIR as a broad screening tool for diabetic kidney disease screening, and several NIR and MIR studies have examined serum or plasma in renal and systemic disease cohorts, including NIR determinations of serum total proteins and urea [14], NIR creatinine estimation in patient blood samples [15], and recent ATR-MIR serum profiling to discriminate CKD on hemodialysis patients from controls [16] and as a screening tool for serum analysis [17]. Comprehensive reviews now synthesize these efforts and emphasize the promise of MIR/NIR as label-free, scalable tools in AKI and CKD, while noting that most applications remain exploratory [18]. Despite these encouraging works, important methodological limitations constrain its direct translational relevance to serum-based CKD diagnostics and as a screening tool for serum analysis.

Besides the limitations associated with the biofluids pointed above, there are also other works with limitations associated with the use of aqueous solutions, or with biofluids where only one or a few target metabolites are varied, not representing the complexity of the disease that leads to the variation of diverse metabolites. For example, Lubnow et al. [4], Ogawa et al. [5], and related aqueous-phase NIR studies exemplify precise optimization of wavelength sets for urea or albumin in controlled media. Shaw [6], Pezzaniti [19] and co-workers used NIR in urine to quantify urea, creatinine, and protein, but within a matrix that lacks the high and structured protein content of serum, while Shaw et al.’s [20] MIR dried-urine film method effectively removes water to facilitate modeling. Henn et al. [11] directly compared MIR and NIR for multicomponent monitoring in dialysate using PLS; however, the target is spent dialysis fluid, again, a less complex and more constrained environment than native serum. Salivary ATR-MIR studies (Rodrigues et al. [12], Lin et al. [21], Tangwanichgapong et al. [22]) and urinary MIR biomarker work (Yu et al. [23]) provide compelling, non-invasive diagnostic and staging performance but operate in matrices and pathophysiological contexts that differ substantially from circulating serum. Even among the relatively few blood/serum-focused examples, most target a single analyte or broad classification rather than simultaneous, regression-grade quantification of the core renal markers within a CKD-relevant design: Hall et al. [14] used NIR for serum proteins and urea but not creatinine; Barnea and Abookasis [15] evaluated NIR for creatinine alone; Perez-Guaita et al. [17] evaluated the use of infrared spectroscopy as a screening tool for serum analysis without specific CKD-stage modeling; Tangwanichgapong et al. [16] recently applied ATR-MIR to serum from hemodialysis patients to identify discriminant bands but did not construct stage-resolved, multi-analyte regression models, like Hall and Pollard [14]. Moreover, many calibration strategies across this literature (including in our own previous work with simple solutions [3]) adopt a “one metabolite at a time” paradigm, where creatinine, urea, or albumin is artificially varied while other CKD-relevant constituents remain fixed. While analytically convenient, such designs do not mirror CKD pathophysiology, in which creatinine and urea increase together as GFR declines and albumin often decreases due to protein-energy wasting, inflammation, or urinary loss, all superimposed upon a complex serum matrix that shapes MIR and NIR spectra through overlapping bands, scattering, and water–protein interaction.

Recent studies (Table 1) have demonstrated that vibrational spectroscopy of biofluids, particularly ATR-MIR of serum or urine extracts, can support CKD/DKD discrimination using chemometrics and machine-learning classifiers, often reporting high diagnostic performance in small to moderate cohorts. However, most published work focuses on a single spectral domain and binary discrimination (disease vs. control), with limited attention given to robust multi-class staging, cross-domain comparison, and systematic benchmarking of classifiers under controlled disease progression conditions. In this context, our study advances the field by combining NIR and MIR spectra with supervised classification to predict both kidney disease presence and disease stage, using simulated disease stages to explicitly probe progression-related spectral signatures and to compare model performance across spectral regions and algorithms.

The present study was conceived to address the gaps pointed above and to move IR-based renal biomarker modeling closer to clinical reality. Here, we extend our previous MIR/NIR comparison by considered serum-based solutions mimicking six phases of kidney function, from healthy profiles to advanced CKD, through the simultaneous and coordinated variation of creatinine, urea, and albumin concentrations in ranges anchored to established CKD staging thresholds. This composed design encodes physiologically plausible covariance between the three biomarkers into both calibration and validation spectral-sets, forcing the chemometric models to learn from realistic multivariate patterns rather than from isolated perturbations. Using these composed serum profiles, we systematically evaluate the performance of MIR and NIR spectroscopy under controlled and complementary measurement conditions: in the MIR region, spectra were acquired from microliter volumes in a 96-well microplate format compatible with minimally invasive sampling and high-throughput analysis; in the NIR region, spectra were acquired directly in aqueous serum using a transflectance fiber optic probe, enabling real-time measurement without dehydration. Building on the harmonized pre-processing and modeling framework introduced in our first paper, we develop regression models for the simultaneous prediction of creatinine, urea, and albumin and classification models to discriminate healthy from diseased profiles and to resolve CKD-like stages. We further interrogate the impact of spectral sub-region selection on both regression and classification performance, with particular emphasis on narrow, information-rich windows that could be targeted by low-cost or miniaturized devices. In contrast to earlier MIR-only, NIR-only, aqueous/urine-based, single-analyte, or dialysate-focused approachesour study provides a dual-platform, multi-marker, stage-mimicking evaluation in a serum matrix expressly designed to reflect CKD progression. This positions the resulting models and the methodological insights on calibration design and spectral windowing as a more clinically relevant foundation for future MIR/NIR-based POC tools for CKD screening and monitoring.

## 2. Materials and Methods

### 2.1. Biological Sample Preparation

A total of 54 solutions were prepared in fetal bovine serum (lote S00AV from Biowest, Nuaillé, France,) at a final dilution of 1/5 (*v*/*v*) in water, where the creatinine, urea and albumin varied simultaneously to mimic various stages of chronic kidney disease (CKD). The 1/5 (*v*/*v*) dilution of fetal bovine serum (FBS) in water was conducted to maximize the ratio of signal to noise in the spectral data, while enabling to add the urea, creatinine and albumin from other concentrated solutions to achieve the final concentrations of those molecules.

**Figure 1:**
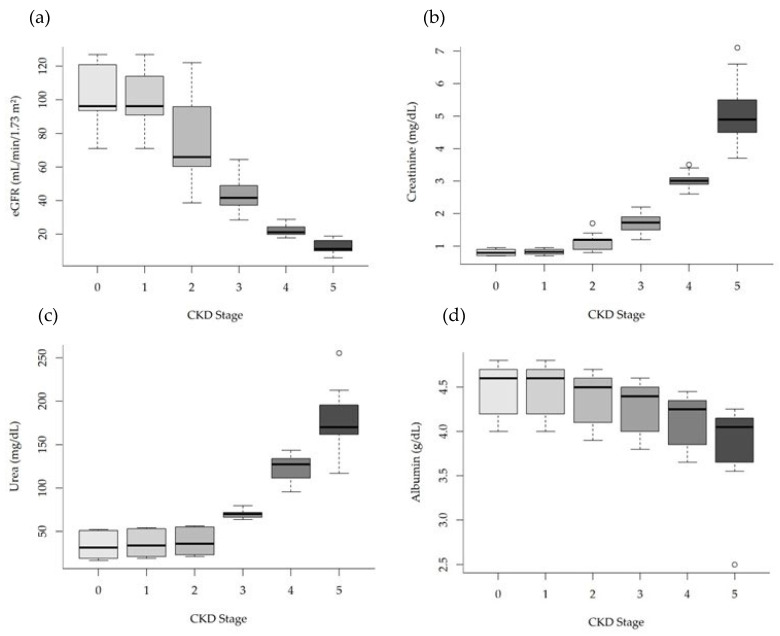
Boxplots of concentration profiles of the estimated glomerular filtration rate (**a**), creatinine (**b**), urea (**c**) and albumin (**d**) of the solutions respective to each CKD stage.

The creatinine (Merck, Darmstadt, Germany) concentrations across CKD stages were based on the 2021 CKD-EPI creatinine equation (eGFRcr), adopted for adult populations [26,27,28]. This equation estimates GFR based on serum creatinine, age, and sex, and is widely used for diagnosing and staging CKD, from stage 1, mild kidney damage, to stage 5, most severe kidney damage (Table 2). A total of 9 solutions of each stage of CKD were prepared. To mimic the diversity of age and gender, it was considered that from the 9 solutions, 5 were from women 25, 34, 43, 50 and 65 years old, while 4 were men 27, 38, 52 and 63 years old (Table 3, Figure 1).

The urea (Merck, Darmstadt, Germany) concentrations in the simulated CKD stages were based on the Blood Urea Nitrogen (BUN) normal range, which is 5 to 20 mg/dL. The BUN is roughly one-half (28/60 or 0.446) of the blood urea [29]. The albumin (Bovine Serum Albumin, lyophilized powder, Sigma-Aldrich, St. Louis, MO, USA) concentration tends to slightly diminish as CKD progresses [30], and the synthesized solutions reflect this overall pattern (Table 3, Figure 1). The 54 solutions were also grouped, from stage 0 to 2, as “healthy”, and between stages 3 and 5 as “diseased” CKD profiles.

### 2.2. MIR-Spectroscopy

First, 20 µL of each solution was placed on IR-transparent Si microtiter plates with 96 wells (Bruker Optics, Ettlingen, Germany) and subsequently dehydrated for 2.5 h in a vacuum desiccator setup (ME2, Vacuubrand, Wertheim, Germany). The MIR spectra were recorded in transmission mode with an HTS-XT associated with a Vertex-70 spectrometer (Bruker Optics), using a spectral resolution of 4 cm^−1^ and 40 scans per sample in the spectral range between 4000 and 400 cm^−1^. Each solution was analyzed in triplicate with a background spectrum obtained from an empty well [3].

### 2.3. NIR-Spectroscopy

Spectra were acquired using an NIR transflection fiber optic probe, IN-271P (Bruker Optics), with a path length of 2 mm, coupled to a Vertex-70 spectrometer (Bruker Optics) by submerging it in 2 mL of each solution. A reference atmospheric air spectrum was acquired before the probe was inserted into the solutions. Spectra were collected in the 12,500–4000 cm^−1^ range, consisting of 32 co-added scans with 8 cm^−1^ resolution. The scanner velocity was set to 10 kHz, and the aperture setting was defined as 6 mm. Background signal removal (blank) of the atmosphere was performed before every sample read.

### 2.4. Spectra Pre-Processing and Processing

Spectra atmospheric compensation was applied to all MIR spectra. Combinations of the following spectral pre-processing methods were evaluated: baseline correction using the Rubber Band method (BC), Standard Normal Variate (SNV), Unit Vector Normalization (UVN), and first (1D) and second (2D) derivatives using a Savitzky–Golay filter with a second-order polynomial and 15 smoothing points [3].

Principal component Analysis (PCA) was conducted for preliminary data patterns search.

Partial Least Squares (PLS) regression models were elaborated with a maximum of 10 latent variables and mean-centered data using the KERNEL PLS algorithm [3]. A total of 162 spectra were considered for MIR analysis, since triplicate spectra were acquired for each solution, and for NIR analysis, an additional 270 spectra were considered, with 5 replica per solution. A cross-validation method was applied, in which 5 cycles of validation were conducted, where for each cycle, 80% of randomly selected samples were used for training and the remaining for validation. The models’ performance was evaluated considering the coefficient of determination (R^2^), the root mean square error (RMSE), and the number of latent variables. To obtain better regression models, defined sub-regions of the MIR and NIR spectrum were evaluated, e.g., the regions associated with the third overtone (12,500–8400 cm^−1^), the second overtone (9100–6250 cm^−1^), and the first overtone (6700–5000 cm^−1^), and the combined band region (5200–4000 cm^−1^).

Supervised classification models of Random Forest (RF), Extreme Gradient Boosting (XGBoost), and Support Vector Machines (SVMs) were implemented to discriminate between healthy and CKD-like serum profiles and to separate samples into the different CKD stages. These models were trained using the same spectral data previously selected for the regression analysis. Model performance was evaluated using random sampling, with 70% of data used for training and 30% for testing. This was repeated 10 times, with stratified sampling applied to preserve class distribution across splits. The model’s performance was assessed using standard classification metrics, namely accuracy, sensitivity, specificity, precision, F1 score, and the area under the ROC curve (AUC).

The SVM model used a radial basis function (RBF) kernel with cost C = 1, and γ computed automatically as 1/k (with k being the number of spectral features). The number of k features of the implemented models were 3720 for the MIR full spectral data, 571 for the MIR 1750–1200 sub-region, 4406 for the NIR full spectral data and 2124 for the NIR third overtone. The RF model was built with 150 trees, a minimum node size of 5 instances and done on a fixed seed. The XGBoost model was implemented with 100 trees, a learning rate of 0.3, maximum tree depth of 6, and L2 regularization with λ = 1, with a fixed seed. All remaining hyperparameters were set in the respective software ware.

Spectra atmospheric compensation and baseline correction were conducted with OPUS^®^ software, version 6.5 (Bruker, Germany), while remaining spectra pre-processing and processing methods were conducted with Unscrambler^®^ X 10.4 software (CAMO software AD, Oslo, Norway).

## 3. Results and Discussion

Based on MIR and NIR spectral data, the following analyses were conducted:

- PCA was performed to enable a preliminary spectral data analysis for a data pattern search.
- PLS regression models were used to predict the creatinine, urea, and albumin concentrations.
- Supervised classification models of Random Forest, XGBoost, and SVM were used to predict the disease, i.e., to discriminate between healthy and diseased states, and the CKD stage.

### 3.1. Data Pattern Search

**Figure 2:**
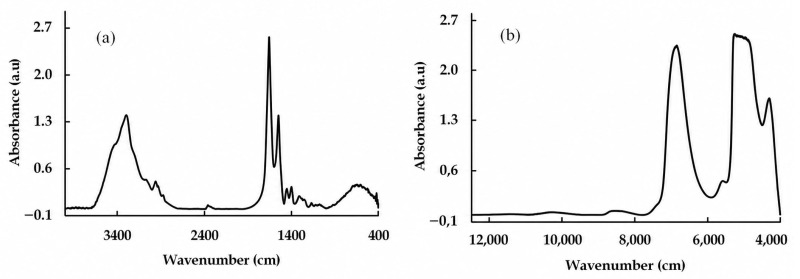

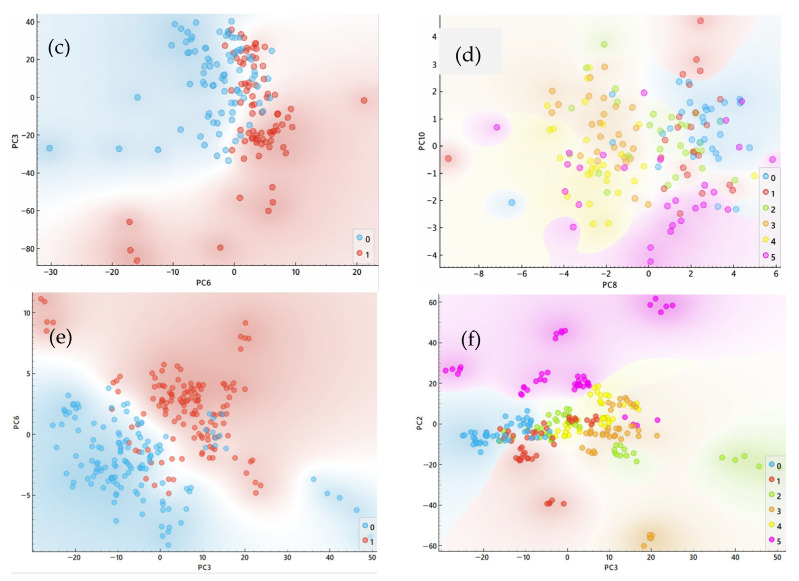
Representations of MIR (**a**) and NIR (**b**) spectra of the CKD mimicking solutions and the corresponding PCAs of spectra (unprocessed) of the MIR (**c**,**d**) and NIR (**e**,**f**) regions with sample separation by “healthy vs. diseased” (**c**,**e**) and by CKD stage (0−5) (**d**,**f**) groups.

Figure 2a,b represent the MIR and NIR spectra of the serum solutions mimicking CKD, with baseline correction, and Figure 2c–f represent the corresponding PCA. Interestingly, the PCA identifies a data pattern, i.e., score separation, according to the “healthy” and “diseased” states, both in the MIR region (Figure 2c), as well as in the NIR region (Figure 2e) of the spectra. A data pattern according to the six phases of CKD can also be observed in the NIR spectra (Figure 2f). These outputs indicate that the spectra provide a molecular fingerprint of CKD stages.

### 3.2. Simultaneous Prediction of the Creatinine, Urea and Albumin Concentrations

The impact of diverse spectra pre-processing methods on the PLS models were evaluated. For the MIR spectral data, baseline correction (BC), normalization (SNV) and 1st and 2nd Savitzky–Golay derivatives (1D and 2D) were evaluated (Table 4). As expected, the validation dataset lead to slightly worse PLS models in relation to the calibration dataset. For the purposes of this work, the models’ performance will be based around the validation dataset.

Based on the whole MIR spectra, it was possible to develop good models to predict the creatinine concentration (R^2^ = 0.76 and RMSE= 0.79 mg/dL) and an excellent model to predict urea (R^2^ = 0.97 and RMSE = 9.98 mg/dL). The best models were built on normalized spectra, after baseline correction or after the first derivative application. It was not possible to develop a model to predict the albumin concentration based on the whole MIR spectra.

Regression models based on sub-regions of the MIR spectra were also developed, based on the normalized first derivative spectra, since in general, this pre-processing method led to better models with the whole spectra (Table 4). It was possible to increase the models’ performance to predict creatinine, since an R^2^ of 0.85 and an RMSE of 0.64 mg/dL were achieved. This model was built in the region between 1200 and 1750 cm^−1^ according to creatinine presenting major spectral bands at 1627 due to C=O, and between 1100 and 1250 cm^−1^ due to C-N bond vibrations [30]. Several of the tested sub-regions led to good regression models for urea prediction, but they were, interestingly, slightly worse than the models based on the whole spectra. These sub-regions, 3500–2800, 1750–1200 and 1450–1200 cm^−1^, are correlated with the N-H vibration of urea, the amide C=O bond and the C-N vibrations, respectively. Although a good regression model for albumin prediction could not be obtained, a more reasonable model was achieved, presenting an R^2^ of 0.71 and an RMSE of 0.22 g/dL for the 1750–1200 cm^−1^ window.

These results align with the existing literature on MIR applications for renal biomarker quantification in serum and other matrices. No serum-based works were found to directly compare with our predicting model of creatinine (R^2^ = 0.85, RMSE = 0.64 mg/L), but other matrices, many of them less complex than serum, have been used for the prediction of creatinine. For example, Henn et al. [11] obtained a creatinine regression model, achieving R^2^ = 0.98 and RMSE = 1.5 mg/dL in dialysate, built with datasets where the creatinine concentration varied a lot more than serum physiological levels (0–33.94 mg/dL). Hoşafçı et al. [31] predicted creatinine in urine, achieving R^2^ = 0.98 and RMSE = 10 mg/dL, based on solutions with a span of 10 to 252 mg/dL. The better performance of these models can be attributed to the simplicity of their respective matrices, as well as the higher range of concentration values, which contrast with the physiological serum creatinine concentrations that were employed in this work (between 0.71 and 7.1 mg/dL). In our previous work [3], we developed excellent models to predict creatinine based on a serum matrix (R^2^ > 0.90), but this was done with samples where only one metabolite’s concentration would vary, which led to models that are not quite as suitable for realistic applications as regression models built with a calibration dataset where all metabolite concentrations are varied simultaneously.

Regarding urea prediction, our best MIR model (R^2^ = 0.97, RMSE = 9.98 mg/dL) also shows comparable performance to the most accurate models reported in the literature. Low-Ying et al. [32] predicted urea concentration in whole blood using dried film MIR spectroscopy over a similar concentration range (15–120 mg/dL versus our 6.4–198.4 mg/dL), achieving R^2^ = 0.94. Jessen et al. [33] developed a regression model for urea prediction in human plasma, reporting an R^2^ = 0.99 and RMSE = 1.5 mg/dL, and Henn et al. [11] also predicted urea concentration from dialysate, achieving R^2^ = 0.99 and RMSE = 6.6 mg/dL. Finally, our best albumin prediction model achieved only moderate performance (R2 = 0.71, RMSE = 0.22 g/dL). This is lower than some reported works: Hoşafçı et al. [31] achieved R^2^ = 0.97 and RMSE = 0.15 g/dL for albumin in blood, while Jessen et al. [33] reported R^2^ = 0.92 and RMSE = 0.6 g/dL for albumin in plasma. The relatively weaker performance for albumin in our study may be attributed to the significantly smaller concentration ranges used across CKD stages compared to creatinine and urea, which both increase dramatically with declining renal function.

The whole NIR spectra led to an adequate model predicting creatinine concentration (Table 5), with an R^2^ of 0.83 and an RMSE of 0.68 mg/dL, like the model obtained with MIR spectroscopy. Using the whole NIR spectra, excellent models were also developed to predict urea and albumin, with both achieving R^2^ higher than 0.90. To further improve some of these models, diverse regression models were developed with sub-regions of the NIR spectra. This made it possible to obtain an excellent model to predict creatinine, based on the third overtone region, achieving an R^2^ of 0.91 and an RMSE of 0.48 mg/dL, better than the values obtained with MIR spectroscopy. The best models to predict urea and albumin concentrations, based on the NIR sub-regions, were as good as those obtained with the whole NIR spectra. The performance of these NIR models also compare favorably with previously reported NIR applications with renal biomarkers. Hall and Pollard [14] were among the first to demonstrate reagent-free NIR determination of total protein, albumin, globulins and urea directly in human serum, reporting for the urea model an R^2^ ≈ 0.99 and a standard error of prediction of approximately 4.8 mg/dL, and an R^2^ = 0.97 and standard error of prediction of 1.1 g/L for albumin. More recently, Barnea and Abookasis [15] used NIR to quantify creatinine in patient blood samples over a 0.96–12.5 mg/dL range and obtained very high agreement with the reference assay, with correlation coefficients above 0.98 and low prediction errors. Despite this, their calibration strategy focused on a single analyte and did not account for the co-variation of other metabolites observed in CKD. In simpler matrices, such as urine, Pezzaniti et al. [19] and Shaw et al. [6] achieved excellent correlation coefficients ≥ 0.99 for urea and creatinine with low standard errors.

Both the present MIR and NIR models are competitive, especially considering that they were developed in a serum matrix where creatinine, urea, and albumin were varied simultaneously to mimic CKD progression rather than being optimized for a single isolated analyte.

**Figure 3:**
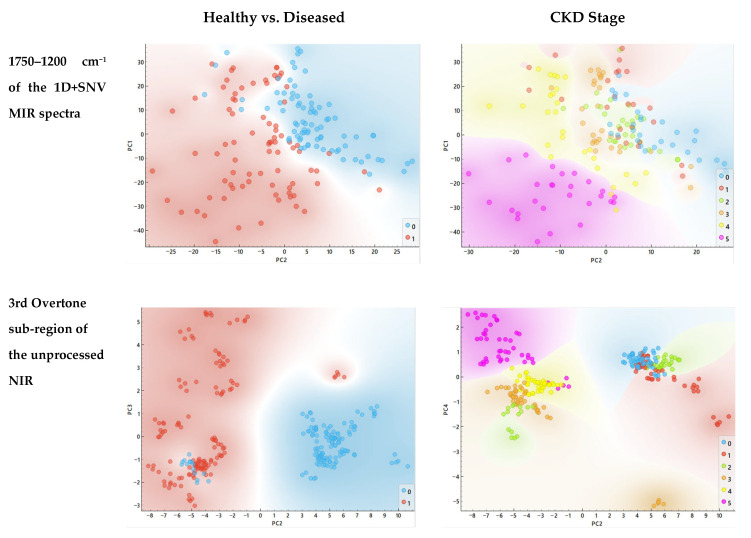
PCA score plot according to healthy vs. diseased (**left** panels) and CKD stage (**right** panels), based on the 1200 to 1750 cm^−1^ region of the normalized first derivative MIR spectra (**top** panels), and the 3rd overtone of the NIR spectra (**bottom** panels).

Since it was possible to maintain or increase the regression models’ performance based on sub-regions on both MIR and NIR spectra, the impact of these sub-regions on the data pattern of the PCA score plot was also evaluated (Figure 3). These PCA were conducted with the corresponding spectra pre-processing methods used by the best regression models, especially to predict creatine, i.e., with normalized first derivative for MIR spectra, and only with baseline correction for the NIR spectra. The score-plot based on the sub-region of the spectra presented data patterns according to healthy vs. diseased and for the CKD stage based on the 1200 to 1750 cm^−1^ sub-region of the MIR spectra and with the 3rd overtone of the NIR region. This points to the possibility of using simpler and cheaper analytical devices for future applications.

### 3.3. Discriminating Disease States and Disease Stages

The supervised classification models Random Forest, XGBoost and SVM were applied. Random Forest models, a bagging ensemble method of independent decision trees, are very robust to overfitting and demonstrate competitive or superior performance to XGBoost across most evaluated conditions. The latter model is based on an advanced gradient boosting algorithm that builds trees sequentially and is usually more complex for hyperparameter tuning. SVM, which is based on finding hyperplanes that maximize the margin between classes, presents advantages for high-dimensional data based on spectra, where the number of features is greater than the number of samples. Detection of chronic kidney disease from routine clinical variables has been addressed using the XGBoost classifier [34]. Other nephrology studies have relied on XGBoost to model CKD risk trajectories and to predict specific complications in patients with advanced CKD, embedding these models in clinical decision support settings [35,36]. Random Forest algorithms have been applied to differentiate glomerular versus tubular patterns of kidney injury using panels of biochemical and histological markers and to construct CKD risk prediction models from demographic, clinical and omics-level data [37,38]. Beyond nephrology, a serum ATR-MIR study in rheumatology employed SVM to separate rheumatoid arthritis, osteoarthritis and healthy controls based solely on spectral fingerprints [38].

In the present work, the three classifiers (Random Forest, XGBoost, SVM) were applied to MIR and NIR spectra to determine the best spectral pre-processing methods and spectral sub-regions obtained in the previous section. For the MIR spectra, the normalized first-derivative spectra were evaluated using the full spectral range and the sub-region between 1750 and 1200 cm^−1^. For the NIR spectra, analyses were performed using baseline correction and the third overtone sub-region.

It was possible to obtain excellent models to discriminate between healthy and diseased states, with AUCs higher or equal to 0.98, for both MIR and NIR spectral data, and for the three classification algorithms (Table 6). These high performances are in agreement with other authors’ work, developing models for renal diseases based on serum spectra, in relation to other types of diseases. For example, serum ATR-MIR models for early gastric cancer detection or for distinguishing rheumatoid arthritis from osteoarthritis reported lower AUC values [38,39] (ranging from 0.72 to 0.90). Zong et al. [40] reported an AUC of 0.94 for detecting CKD using serum with Surface-Enhanced Raman Scattering (SERS), while Tangwanichgapong et al. [16], in a recent serum ATR-MIR study, distinguished end-stage renal disease from healthy controls, achieving perfect classification metrics (100% sensitivity/specificity), and confirming that advanced renal failure imprints a defined spectral signature on serum.More notably, however, is the ability of the present models to accurately stratify samples into the six distinct CKD-like stages, with AUC higher or equal to 0.93 for MIR and higher or equal to 0.97 for NIR, with the three classification models. Differentiating early-to-moderate CKD stages is analytically challenging due to the smaller changes in biomarker concentrations. Zong et al. [40] achieved total accuracy of only 78% when classifying clinical CKD stages using serum analyzed with SERS, noting that in their work, they only distinguished between three major groups, those being healthy (equivalent to our stages 0–1, stages 2–3 and stages 4–5). Similarly, Marom et al. [41] analyzed breath with gold nanoparticle sensors and achieved only 79% accuracy in distinguishing early-stage CKD from healthy states, improving to 85% for advanced stages. In our work, and especially with the NIR models, excellent models with an AUC higher or equal to 0.97 enabled discrimination between the six CKD phases. This performance is comparable to models using more complex data, such as a Random Forest model based on ultrasound radiomics to predict renal fibrosis in CKD, which achieved AUCs of 0.93–0.96 [42], and expert systems integrating multiple clinical variables (e.g., ANN/XGBoost on laboratory data), which report similar high-performance metrics for CKD stage prediction/classification [43,44]. Table 7 displays a quick summary of the scientific articles mentioned above.

## 4. Conclusions

Due to the high prevalence and health impact of CKD, it is relevant to develop new analytical procedures to be implemented in a simple, economic mode, enabling a frequent monitoring procedure at clinical set-ups and as point-of-care devices. The vibrational spectroscopic techniques based either on the NIR or the MIR region, present characteristics that may enable us to achieve those goals. In the present work, NIR and MIR spectroscopy were applied to serum samples mimicking the 6 stages of CKD, by simultaneously varying creatinine, urea and albumin. It was evaluated diverse spectra pre-processing techniques on the development of regression models to simultaneously predict the concentration of these three biomarkers, as well to discriminate, based on supervised classification methods, healthy vs. diseased stage, and the six stages of the disease. It was also evaluated the use of sub-regions of these spectral regions to evaluate future applications using simpler and low-cost equipment’s at clinical institutions or even at point-of-care devices.

The MIR region enabled the best predicting model of the urea concentration, with R^2^ of 0.97 and a RMSE of 10.0 mg/dL but enabled the development of only a good model to predict creatinine, with an R^2^ of 0.85 and an RMSE of 0.64 mg/dL and did not enable the development of predicting models of albumin. The NIR region, including its sub-region corresponding to the 3rd overtone, enabled the development of excellent models for the three biomarkers, all with R^2^ higher than 0.90. The models to predict creatinine and albumin were much better than the ones based on the MIR spectra, while the model to predict urea was also excellent, with an R^2^ 0.91 and an RMSE of 16.8 mg/dL, in relation to the best achieved by MIR spectra with R^2^ of 0.96 and a RMSE of 12.0 mg/dL.

Both MIR and NIR spectra, including the evaluated sub-regions, enabled the development of excellent classification models, discriminating healthy from diseased stages, with an AUC of 0.99, and recall and specificity values higher than 0.98.

The best models to discriminate among the six phases of CKD also presented AUCs of 0.94 and 0.99 for the MIR and NIR regions, respectively, with the best model achieved by the whole NIR spectra, with an AUC of 0.99, a recall of 0.87 and a specificity of 0.97.

Overall, these findings highlight MIR and NIR spectroscopy as relevant analytical techniques to be explored for a low-cost solution for intensive monitoring of CKD. Due to the complementary nature of the techniques, both should be evaluated concerning the goal of the analysis. MIR enables high-throughput analysis based on microliter volume samples, needing only a simple dehydration step. The NIR region can enable analysis based on lower-cost portable equipment, especially if a sub-region of the NIR spectra is used. Both scenarios offer characteristics that should be further explored for more efficient monitoring, and consequently, control of CKD.

This “proof-of-concept”, based on the simulation of CKD, by simultaneously varying the three critical molecules, i.e., creatinine, urea and albumin in serum, should be validated in the future using real patient cohorts. This will enable the incorporation of potential cofound variables such as age and sex and from CKD, such as accumulated uremic toxins, co-prescribed drugs, lipids, inflammatory mediators among others since, e.g., CKD patients present a high prevalence of diabetic nephropathy and dyslipidemia, with hypertriglyceridemia affecting 45–68% of patients across stages 1–4 [45].

## Figures and Tables

**Table 1:** Recent studies applying MIR and NIR spectroscopy to kidney disease diagnosis and staging.

Reference	Matrix	Spectral Domain	Task (Binary/Multi)	n (Classes)	Best Reported Metrics (Primary Set)
Tangwanichgapong et al. [16] (2025)	Serum	ATR-MIR	ESRD (hemodialysis) vs. control (binary)	42 (21/21)	PLS-DA: Acc 1.00; Sens 1.00; Spec 1.00
Navarro-Esteve et al. [13] (2025)	Urine protein extracts	ATR-MIR	DKD vs. control (binary) and microalbuminuria identification (binary)	AUS: 177 (155 DKD/22 ctrl); ESP: 61 (35 DKD/26 ctrl)	PLS-DA: AUC 0.87 (DKD); SVMDA: AUC 0.98 (microalbuminuria)
Khanmohammadi et al. [24] (2013)	Serum	ATR-MIR, 1800–900 cm^−1^	Renal failure vs. normal (binary)	75 (35 RF/40 normal)	SIMCA Acc 0.95; Sens 1.00; Spec 0.91
Ramalhete et al. [25] (2025)	Serum (pre-biopsy)	MIR	(1) Rejection vs. non-rejection (binary) (2) TCMR vs. AMR (binary)	81 (25 non-rej/56 rej; within rej: 12 TCMR/44 AMR)	Naïve Bayes (1) AUC 0.95 (2) AUC 0.99

**Table 2:** Serum creatinine concentration across CKD stages, as determined by the eGFR.

Stage of CKD	GFR Interval	Corresponding Creatinine Interval (mg/dL)		Implication
		Minimum	Maximum	
Stage 1	>90	-	1.07	Mild kidney damage Kidney works as well as normal
Stage 2	60–89	1.08	1.50	Mild kidney damage Kidney still works well
Stage 3a	45–59	1.52	1.9	Mild to moderate kidney damage Kidneys do not work as well as they should
Stage 3b	30–44	1.95	2.7	Moderate to severe damage Kidneys do not work as well as they should
Stage 4	15–29	2.75	4.7	Severe kidney damage Kidney is close to not working at all
Stage 5	<15	4.9	-	Most severe kidney damage Kidneys are very close to not working or have stopped working (failed)

**Table 3:** Physiological and biochemical makeup of the 54 solutions that mimic renal function in healthy individuals (stage 0) and across multiple degrees of renal impairment (stages 1–5).

CKD Stage	Age	25	27	34	38	40	43	52	65	63
	Gender	F	M	F	M	F	F	M	F	M
0	Creatinine (mg/dL)	0.71	0.75	0.90	0.70	0.80	0.80	0.95	0.90	0.70
	Urea (mg/dL)	17	19	32	51	32	34	19	51	52
	Albumin (g/dL)	4.8	4.2	4.1	4.6	4	4.5	4.6	4.8	4.7
	eGFR ((mL/min/1.73 m^2^)-CKD-EPI 2021)	120.9	126.8	86.0	121.0	95.5	93.5	96.3	70.9	103.5
1	Creatinine (mg/dL)	0.71	0.75	0.90	0.85	0.8	0.82	0.95	0.90	0.70
	Urea (mg/dL)	19	21	34	53	34	36	21	53	54
	Albumin (g/dL)	4.8	4.2	4.1	4.6	4	4.5	4.6	4.8	4.7
	eGFR ((mL/min/1.73 m^2^)-CKD-EPI 2021)	120.9	126.8	86.0	114.1	95.5	91.0	96.3	70.9	103.5
2	Creatinine (mg/dL)	0.8	0.9	1.2	1.4	1.7	1.2	1.2	1.2	0.9
	Urea (mg/dL)	21.3	23.4	36.2	55.3	36.2	38.4	23.4	55.3	56.4
	Albumin (g/dL)	4.6	4.1	4.0	4.5	3.9	4.4	4.5	4.7	4.6
	eGFR ((mL/min/1.73 m^2^)-CKD-EPI 2021)	104.8	122.1	60.9	66.0	38.6	60.2	72.8	50.2	96.0
3	Creatinine (mg/dL)	1.2	1.9	1.7	1.5	2.2	1.7	1.8	1.4	2.1
	Urea (mg/dL)	63.8	65.2	66.5	69.1	71.8	70.3	74.5	71.8	79.8
	Albumin (g/dL)	4.5	4.0	3.9	4.4	3.8	4.3	4.4	4.6	4.5
	eGFR ((mL/min/1.73 m^2^)-CKD-EPI 2021)	64.4	49.0	40.1	60.7	28.4	37.3	44.7	41.8	34.7
4	Creatinine (mg/dL)	3.5	3.4	2.9	3.1	2.9	3.0	2.6	2.6	3.1
	Urea (mg/dL)	127.7	111.7	111.7	143.6	95.7	124.5	127.7	134.0	143.6
	Albumin (g/dL)	4.4	3.9	3.8	4.3	3.7	4.1	4.3	4.5	4.4
	eGFR ((mL/min/1.73 m^2^)—CKD-EPI 2021)	17.8	24.4	21.1	25.4	20.4	19.1	28.8	19.9	21.8
5	Creatinine (mg/dL)	5.5	4.5	4.9	4.5	4.6	5.2	3.7	7.1	6.6
	Urea (mg/dL)	170.2	161.7	195.7	212.8	117.0	180.3	255.3	159.6	170.2
	Albumin (g/dL)	4.2	3.7	3.6	4.1	2.5	3.8	4.1	4.3	4.2
	eGFR ((mL/min/1.73 m^2^)—CKD-EPI 2021)	10.4	17.4	11.3	16.3	11.7	10.0	18.8	5.9	8.8

**Table 4:** PLS models based on the whole MIR spectra according to diverse pre-processing methods, with the sub-region analysis of the best pre-processing method (first derivative normalized spectra). RMSE values are present in mg/dL for creatinine and urea and g/dL for albumin. The best models are highlighted in bold.

Metabolite	Pre-Processing/Sub-Region	Latent Variables	Calibration		Validation	
			R^2^	RMSE	R^2^	RMSE
**Creatinine**	Raw	8	0.77	0.77	0.72	0.86
	BC	6	0.76	0.80	0.71	0.88
	**BC+SNV**	5	**0.79**	**0.74**	**0.76**	**0.80**
	1D	3	0.76	0.80	0.71	0.88
	**1D+SNV**	3	**0.81**	**0.71**	**0.76**	**0.79**
	2D	7	0.91	0.48	0.69	0.92
	2D+SNV	6	0.92	0.45	0.68	0.93
**Urea**	Raw	8	0.95	12.82	0.92	16.08
	BC	7	0.94	14.39	0.90	17.90
	**BC+SNV**	**6**	**0.98**	**8.69**	**0.97**	**9.98**
	1D	4	0.94	13.76	0.90	17.68
	**1D+SNV**	**4**	**0.98**	**8.50**	**0.97**	**10.12**
	2D	7	0.97	9.67	0.87	20.70
	2D+SNV	7	0.97	8.94	0.91	17.55
**Albumin**	Raw	7	0.52	0.28	0.44	0.30
	BC	6	0.52	0.28	0.46	0.30
	BC+SNV	5	0.55	0.27	0.49	0.29
	**1D**	**10**	**0.90**	**0.13**	**0.53**	**0.28**
	**1D+SNV**	**10**	**0.92**	**0.11**	**0.52**	**0.28**
	2D	1	0.25	0.35	0.23	0.36
	2D+SNV	1	0.43	0.31	0.28	0.35
**Normalized First Derivative (1D+SNV) sub-regions of the spectra (cm^−1^)**						
**Creatinine**	3500–2800	3	0.79	0.75	0.75	0.82
	3500–3200	3	0.79	0.74	0.75	0.82
	3200–3000	3	0.74	0.83	0.68	0.93
	3000–2800	4	0.60	1.03	0.51	1.15
	**1750–1200**	**9**	**0.92**	**0.45**	**0.85**	**0.64**
	1750–1600	4	0.79	0.74	0.77	0.78
	1600–1500	3	0.79	0.75	0.78	0.78
	1450–1200	3	0.79	0.75	0.77	0.79
	1200–1000	4	0.82	0.70	0.74	0.83
	1200–600	3	0.81	0.72	0.74	0.84
**Urea**	3500–2800	4	0.96	10.84	0.96	12.02
	3500–3200	3	0.96	11.62	0.95	12.29
	3200–3000	5	0.88	20.11	0.82	24.48
	3000–2800	6	0.64	34.50	0.46	42.43
	1750–1200	3	0.96	11.13	0.96	11.58
	1750–1600	4	0.96	11.92	0.95	12.55
	1600–1500	4	0.96	11.39	0.95	12.84
	**1450–1200**	**3**	**0.97**	**10.56**	**0.96**	**11.37**
	1200–1000	3	0.94	13.49	0.93	15.05
	1200–600	5	0.96	11.02	0.92	15.90
**Albumin**	3500–2800	3	0.55	0.27	0.48	0.30
	3500–3200	2	0.51	0.28	0.47	0.30
	3200–3000	4	0.60	0.26	0.45	0.30
	3000–2800	3	0.47	0.30	0.37	0.33
	**1750–1200**	**8**	**0.80**	**0.18**	**0.71**	**0.22**
	1750–1600	8	0.66	0.24	0.56	0.27
	1600–1500	1	0.44	0.30	0.42	0.31
	1450–1200	5	0.64	0.24	0.55	0.27
	1200–1000	3	0.45	0.30	0.33	0.34
	1200–600	5	0.75	0.20	0.44	0.30

**Table 5:** PLS models based on the whole NIR spectra according to diverse pre-processing methods, with the sub-region analysis of the best pre-processing method (baseline correction). RMSE values are present in mg/dL for creatinine and urea and g/dL for albumin. The best models are highlighted in bold.

Metabolite	Pre-Processing/Sub-Region	Latent Variables	Calibration		Validation	
			R^2^	RMSE	R^2^	RMSE
**Creatinine**	**Raw**	**5**	**0.91**	**0.50**	**0.83**	**0.68**
	BC	5	0.88	0.56	0.73	0.85
	BC+SNV	5	0.84	0.66	0.61	1.02
	1D	10	0.92	0.45	0.38	1.28
	1D+SNV	10	0.92	0.46	0.36	1.32
	2D	1	0.25	1.41	0	1.72
	2D+SNV	1	0.24	1.42	0	1.76
**Urea**	**Raw**	**7**	**0.97**	**9.27**	**0.91**	**17.05**
	BC	7	0.97	9.49	0.86	21.26
	BC+SNV	6	0.93	15.60	0.72	30.72
	1D	10	0.91	16.97	0.29	48.50
	1D+SNV	10	0.91	17.03	0.26	49.70
	2D	1	0.26	49.50	0	62.20
	2D+SNV	1	0.25	49.73	0	63.20
**Albumin**	Raw	4	0.94	0.10	0.92	0.11
	**BC**	**4**	**0.94**	**0.10**	**0.93**	**0.11**
	BC+SNV	4	0.92	0.11	0.90	0.13
	1D	7	0.95	0.09	0.83	0.17
	1D+SNV	7	0.95	0.09	0.81	0.18
	2D	1	0.25	0.35	0	0.46
	2D+SNV	1	0.25	0.35	0	0.46
**Baseline-corrected sub-regions of the spectra (cm^−1^)**						
**Creatinine**	**3rd Overtone**	**6**	**0.98**	**0.24**	**0.91**	**0.48**
	2nd Overtone	4	0.59	1.04	0.53	1.12
	1st Overtone	2	0.44	1.22	0.41	1.26
	Combination Bands	4	0.79	0.74	0.55	1.10
	11,000–8600	6	0.95	0.37	0.84	0.65
	7800–7050	10	0.80	0.73	0.70	0.90
	5800–5300	10	0.76	0.80	0.71	0.88
**Urea**	**3rd Overtone**	**6**	**0.97**	**8.80**	**0.91**	**16.84**
	2nd Overtone	10	0.85	22.44	0.49	41.22
	1st Overtone	2	0.32	47.23	0.29	48.80
	Combination Bands	4	0.82	24.62	0.58	37.19
	11,000–8600	7	0.96	11.06	0.84	22.94
	7800–7050	10	0.79	26.46	0.67	32.84
	5800–5300	10	0.80	25.56	0.77	27.61
**Albumin**	3rd Overtone	7	0.97	0.07	0.81	0.18
	**2nd Overtone**	**4**	**0.94**	**0.10**	**0.93**	**0.11**
	**1st Overtone**	**4**	**0.94**	**0.10**	**0.93**	**0.11**
	Combination Bands	7	0.96	0.08	0.80	0.18
	11,000–8600	5	0.89	0.13	0.71	0.22
	7800–7050	6	0.92	0.12	0.89	0.14
	5800–5300	8	0.94	0.10	0.93	0.11

**Table 6:** Performance metrics (AUC, recall, specificity, accuracy, precision, and F1-score) of the best discriminant models for classifying healthy vs. diseased profiles and CKD stages based on MIR and NIR spectra. The best models are highlighted in bold.

Discriminant Parameter	Region	Spectrum	Tested Models	AUC	Recall	Specificity	Accuracy	Precision	F1
**Healthy vs. Diseased**	**MIR**	Raw whole spectra	XGBoost	0.94	0.87	0.87	0.87	0.87	0.87
			SVM	0.77	0.68	0.68	0.68	0.69	0.68
			Random Forest	0.92	0.84	0.84	0.84	0.84	0.84
		1D+SNV whole spectra	**XGBoost**	**0.99**	**0.96**	**0.96**	**0.96**	**0.96**	**0.96**
			SVM	0.98	0.93	0.93	0.93	0.93	0.93
			Random Forest	0.99	0.97	0.97	0.97	0.97	0.97
		1D+SNV (1750–1200)	XGBoost	0.99	0.95	0.95	0.95	0.95	0.95
			SVM	0.99	0.94	0.94	0.94	0.94	0.94
			Random Forest	0.99	0.94	0.94	0.94	0.94	0.94
	**NIR**	Raw whole spectra	XGBoost	0.99	0.94	0.94	0.94	0.94	0.94
			SVM	1.00	0.95	0.96	0.95	0.96	0.95
			Random Forest	1.00	0.98	0.98	0.98	0.98	0.98
		BC whole spectra	**XGBoost**	**0.98**	**0.93**	**0.93**	**0.93**	**0.93**	**0.93**
			SVM	1.00	0.96	0.96	0.96	0.96	0.96
			Random Forest	0.99	0.95	0.95	0.95	0.95	0.95
		BC (3rd Overtone)	XGBoost	0.97	0.93	0.92	0.93	0.93	0.93
			SVM	0.94	0.92	0.92	0.92	0.93	0.92
			Random Forest	0.99	0.95	0.95	0.95	0.95	0.95
**Stage**	**MIR**	Raw whole spectra	XGBoost	0.81	0.46	0.89	0.46	0.46	0.46
			SVM	0.69	0.35	0.87	0.35	0.34	0.32
			Random Forest	0.82	0.46	0.89	0.46	0.44	0.44
		1D+SNV whole spectra	XGBoost	0.89	0.65	0.93	0.65	0.64	0.64
			SVM	0.88	0.54	0.91	0.54	0.54	0.52
			Random Forest	0.93	0.68	0.93	0.68	0.66	0.66
		1D+SNV (1750–1200)	XGBoost	0.92	0.72	0.95	0.72	0.71	0.71
			**SVM**	**0.93**	**0.72**	**0.94**	**0.72**	**0.72**	**0.72**
			Random Forest	0.94	0.74	0.94	0.74	0.74	0.74
	**NIR**	Raw whole spectra	XGBoost	0.96	0.76	0.95	0.76	0.77	0.76
			SVM	0.96	0.71	0.94	0.71	0.72	0.70
			**Random Forest**	**0.99**	**0.87**	**0.97**	**0.87**	**0.88**	**0.87**
		BC whole spectra	XGBoost	0.97	0.82	0.96	0.82	0.82	0.82
			SVM	0.98	0.83	0.97	0.83	0.83	0.83
			Random Forest	0.97	0.82	0.96	0.82	0.82	0.82
		BC (3rd Overtone)	XGBoost	0.93	0.68	0.94	0.68	0.69	0.68
			SVM	0.88	0.51	0.90	0.51	0.61	0.50
			Random Forest	0.96	0.77	0.95	0.77	0.78	0.77

**Table 7:** Comparison of the diagnostic performance obtained in the present work with other reported spectroscopic and machine learning methods for CKD detection and staging.

Scientific Article	Methodology	Matrix	Biomarkers	Healthy vs. Diseased	CKD Stage Separation	Observations
Present Work	MIR/NIR	Serum	Creatinine, urea, albumin	AUC > 0.97	6 stages AUC > 0.93 (MIR) AUC > 0.98 (NIR)	High-throughput (MIR) or in situ (NIR) analysis. Physiologically CKD-like calibration of the 5 stages.
Zong et al. [40]	SERS	Serum, Urine	Creatinine, urea	AUC > 0.94 (Serum) AUC > 0.89 (Urine)	Accuracy = 78% Overlap of CKD stages (0–1, 2–3, 4–5)	Good binary classification (healthy vs. diseased). Lower resolution of CKD stages with moderate accuracy.
Marom et al. [41]	Gold nanoparticle sensors	Breath	Volatile organic compounds	Accuracy = 79% (Early CKD vs. Healthy)	Accuracy of 79 and 85% for stages 0–1 to 2–3 and 4 to 5, respectively	Limited accuracy for early disease compared to serum methods.
Rodrigues et al. [12]	ATR-FTIR	Saliva	Thioisocyanate, phospholipids, carbohydrates	AUC ~ 0.88	N/A	Saliva is less invasive but offers lower discriminatory power than serum for this condition.
Tangwanichgapong et al. [16]	ATR-FTIR	Serum	Lipids, proteins, etc.	Accuracy = 100%	N/A	Excellent for End-Stage Renal Disease (ESRD), but did not test stratification of earlier stages.
Metherall et al. [43]	Clinical Data	N/A	Clinical markers	AUC > 0.96	N/A	Successful application of ML algorithms to diverse sets of clinical data (at home, laboratory and monitoring) for the binary classification of healthy vs. diseased profiles.

## Data Availability

The data presented in this study will be available on request from the corresponding author.
